# From global to regional and back again: common climate stressors of marine ecosystems relevant for adaptation across five ocean warming hotspots

**DOI:** 10.1111/gcb.13247

**Published:** 2016-03-21

**Authors:** Ekaterina Popova, Andrew Yool, Valborg Byfield, Kevern Cochrane, Andrew C. Coward, Shyam S. Salim, Maria A. Gasalla, Stephanie A. Henson, Alistair J. Hobday, Gretta T. Pecl, Warwick H. Sauer, Michael J. Roberts

**Affiliations:** ^1^ National Oceanography CentreU. Southampton Waterfront CampusSouthamptonSO14 3ZHUK; ^2^ Rhodes UniversityDrosty RoadGrahamstown6139South Africa; ^3^ Central Marine Fisheries Research InstitutePost Box No. 1603Ernakulam North P.O.Kochi‐682 018India; ^4^ Fisheries Ecosystems LaboratoryOceanographic InstituteUniversity of São PauloCidade UniversitáriaSão PauloSP05580‐120Brazil; ^5^ Oceans and Atmosphere FlagshipCSIROHobartTAS7000Australia; ^6^ Institute for Marine and Antarctic StudiesUniversity of TasmaniaPO Box 49HobartTAS7001Australia

**Keywords:** boundary currents, climate change, ecosystems, marine hotspots, modelling, ocean

## Abstract

Ocean warming ‘hotspots’ are regions characterized by above‐average temperature increases over recent years, for which there are significant consequences for both living marine resources and the societies that depend on them. As such, they represent early warning systems for understanding the impacts of marine climate change, and test‐beds for developing adaptation options for coping with those impacts. Here, we examine five hotspots off the coasts of eastern Australia, South Africa, Madagascar, India and Brazil. These particular hotspots have underpinned a large international partnership that is working towards improving community adaptation by characterizing, assessing and projecting the likely future of coastal‐marine food resources through the provision and sharing of knowledge. To inform this effort, we employ a high‐resolution global ocean model forced by Representative Concentration Pathway 8.5 and simulated to year 2099. In addition to the sea surface temperature, we analyse projected stratification, nutrient supply, primary production, anthropogenic CO_2_‐driven ocean acidification, deoxygenation and ocean circulation. Our simulation finds that the temperature‐defined hotspots studied here will continue to experience warming but, with the exception of eastern Australia, may not remain the fastest warming ocean areas over the next century as the strongest warming is projected to occur in the subpolar and polar areas of the Northern Hemisphere. Additionally, we find that recent rapid change in SST is not necessarily an indicator that these areas are also hotspots of the other climatic stressors examined. However, a consistent facet of the hotspots studied here is that they are all strongly influenced by ocean circulation, which has already shown changes in the recent past and is projected to undergo further strong change into the future. In addition to the fast warming, change in local ocean circulation represents a distinct feature of present and future climate change impacting marine ecosystems in these areas.

## Introduction

Footprints of climate change have been reported for nearly all major marine ecosystems around the world (e.g. Hoegh‐Guldberg & Bruno, 2010; Hobday & Lough, 2011; Wassmann *et al*., 2011; Okey *et al*., 2014). However, neither the physical drivers of climate change nor their impacts on ocean ecosystems manifest homogeneously over the world oceans. For instance, waters of subtropical western boundary currents are warming two to three times faster than the global mean for the world's oceans (Wu *et al*., 2012). Elsewhere, polar amplification leads to Arctic Ocean warming faster than the global trend (e.g. Pithan & Mauritsen, 2014). Such amplification, together with the associated retreat of Arctic sea ice, leads to ecosystem changes that are often in the opposite direction to global trends, with primary production increasing rather than following the global decline (e.g. Popova *et al*., 2014). Meanwhile, the highly productive upwelling zones of eastern boundary currents have a strong sensitivity to climate change, driven by changing patterns of upwelling events that are becoming less frequent but stronger, and longer in duration (e.g. Iles *et al*., 2012).

On the basis of historical observations of sea surface temperature (SST), Hobday & Pecl (2014; henceforth HP14) identified 24 fast‐warming marine areas – so‐called hotspots – and suggested that these could serve as ‘natural laboratories’ where the mechanistic links between ocean warming and biological responses could be studied in advance of wider scale impacts predicted for later in the 21st century. Furthermore, climate adaptation options in marine hotspots could be explored as human dependence on marine resources is very high in many of these areas. During the 21st century, changes in ocean physical and biogeochemical parameters are anticipated to greatly impact ocean ecosystems. Coastal‐marine food resources will alter as a result of species‐specific direct responses to drivers of climate change, such as distribution and abundance of species changing in response to temperature, as already reported from south‐east Australia (Frusher *et al*., 2014), or ocean acidification in the Arctic (e.g. Mathis *et al*., 2015). Such impacts to living marine resources will require individuals, communities, industries and governments to understand and adapt to the changing climate (e.g. Barange *et al*., 2014; Frusher *et al*., 2014). However, adaptation options within the context of climate change must build on a solid understanding of the physical, biological and human aspects of the given systems, and a recognition that marine systems and human societies are really parts of a unified marine socio‐ecological system (Perry *et al*., 2010).

However, rising temperatures are not the only climatic factor impacting ocean ecosystems. ‘Warming up, turning sour, losing breath’ (Gruber, 2011) has become a widely used summary of the major climatic stressors of ocean ecosystems: warming, acidification and deoxygenation, all with implications for marine productivity (Doney *et al*., 2012; Bopp *et al*., 2013). Changing ocean stratification and circulation may also provide wide‐ranging biological effects (Doney *et al*., 2012). Changes in these climatic factors are driven by different mechanisms and different aspects of global ocean dynamics and biogeochemistry (Bopp *et al*., 2013), and consequently, patterns of their fastest changes (or hotspots) do not necessarily coincide in space. Although warming of the ocean may not always be the strongest climatic factor affecting marine ecosystems (e.g. Marañón *et al*., 2014), the rise of the SST probably remains the most unequivocal signature of the climate change. Thus, in this study, we begin with the framework of SST hotspots suggested by Hobday & Pecl (2014). We closely examine five marine SST‐defined hotspots that affect areas off the coasts of Eastern Australia, Brazil, South Africa, India and Madagascar and investigate whether other climatic stressors of marine ecosystem are likely to manifest themselves in these areas. These particular hotspots have marine resource‐dependent communities and provide examples of social, economic and ecological commonalities and contrasts that are a focus of a large international partnership working towards reducing coastal vulnerability (Hobday *et al*., 2016). This partnership, ‘Global understanding and learning for local solutions: Reducing vulnerability of marine‐dependent coastal communities’ (GULLS), works towards improving community adaptation efforts by characterizing, assessing and projecting the likely future of coastal‐marine food resources through the provision and sharing of knowledge between regional hotspots. In order to provide a unifying tool to assess climate change mechanisms common across these temperature‐driven hotspots and to quantify changes in stratification, ocean circulation, nutrient supply, primary productivity, acidification and deoxygenation, we use a global ocean biogeochemical model coupled to a climate model. Crucially, this model is at higher resolution than those used in IPCC AR5 which allows much greater regional realism.

## Materials and methods

### High‐resolution ocean projection

Our ocean projection uses the framework of the Nucleus for European Modelling of the Ocean (NEMO) model. This is comprised of an ocean general circulation model, OPA (Madec, 2008), coupled with a sea‐ice model, LIM2 (Timmermann *et al*., 2005). NEMO version 3.5 is used here with a horizontal resolution of approximately 1/4° and a vertical grid of 75 levels increasing from 1 m thickness at the surface to 200 m at abyssal depths. Vertical mixing is parameterized using the turbulent kinetic energy scheme of Gaspar *et al*. (1990) and includes modifications from Madec (2008).

Biogeochemistry in NEMO is represented by the plankton ecosystem model MEDUSA‐2 (Yool *et al*., 2013a,b). This is a size‐based, intermediate complexity model that divides the plankton community into ‘small’ and ‘large’ portions, and which resolves the elemental cycles of nitrogen, silicon, iron, carbon and oxygen. NEMO is forced at the surface here using output from a simulation of the HadGEM2‐ES Earth system model run by the UK Meteorological Office (UKMO) which includes representations of the terrestrial and oceanic carbon cycles, atmospheric chemistry and aerosols (Collins *et al*., 2011). This HadGEM2‐ES simulation (Jones *et al*., 2011) was performed as part of the Coupled Model Intercomparison Project 5 (CMIP5) and Intergovernmental Panel on Climate Change (IPCC) Assessment Report 5 (AR5). The model's physical ocean state was initialized from the same initial state as HadGEM2‐ES; biogeochemistry was initialized using World Ocean Atlas (nutrients and oxygen) and GLODAP (DIC and alkalinity) climatology products.

To decrease the computational cost of the simulation, the 1/4° model was initialized in 1975 using an initial condition derived from a 1° ‘twin’ spun‐up from 1860 to 1975 under the same forcing data set. Intercomparison of the two model runs in an overlap period (1975–2000) found that they agreed well across a broad range of physical (temperature and salinity) and biogeochemical (nutrients, phyto‐ and zooplankton and primary production) metrics (Yool *et al*., 2015). Nonetheless, during this interval, the models diverged in certain regions (e.g. equatorial upwelling zones, Southern Ocean) where the increased resolution of the 1/4° model permitted improved performance (e.g. mesoscale features, vertical physics).

Further details of model implementation, forcing, equilibration and verification can be found in Yool *et al*. (2013a,b, 2015) and Popova *et al*. (2010, 2014). The model shows good skill in reproducing main features of ocean dynamics and biogeochemistry, and in particular, an improvement in the representation of the main drives of the upper ocean biogeochemistry and ecosystems including upper ocean mixing and circulation.

The relative deviation of the decadal‐average model characteristics of 2050–2059 (‘2050s’) from that of the period 2000–2010 (‘2000s’) was calculated as:

$$X_{2050s}-X_{2000s}/X_{2000s}$$

### Observational data sets

Key aspects of our analysis are based on ocean productivity and circulation, and these require validation with observational products. Following Yool *et al*. (2013a,b), observed primary production is based on the simple average of three satellite‐derived estimates, the VGPM (Behrenfeld & Falkowski, 1997), Eppley‐VGPM (Carr *et al*., 2006) and CbPM (Westberry *et al*., 2008) techniques. To assess the realism of large‐scale surface current patterns, we compare model output with absolute geostrophic velocities derived from satellite altimetry. The specific altimeter products used were produced by Ssalto/*Duacs* and distributed by AVISO, with support from CNES (http://www.aviso.oceanobs.com/duacs). Large‐scale surface currents are in near geostrophic balance, and satellite altimetry provides an accurate estimate of their position. We note that in addition to the geostrophic velocities derived from the slope in sea surface height (SSH) in AVISO, surface velocities also contain an ageostrophic component (e.g. Ekman velocity). However, the discrepancies between the actual and the geostrophic surface velocities are small and do not affect the validity of using geostrophic velocities derived from altimetry to assess the pathways of surface ocean currents in our model.

### Climatic stressors: baseline variability vs. trends

Climatic factors affecting marine ecosystems, such as SST, are expected to show a climate change‐driven trend against a background of interannual variability. In analysing their future changes, the key question we address is whether the trend is equally substantial across all hotspots and, if so, how can we characterize potential significance for ecosystems? We suggest that the answer most relevant to the development, implementation and evaluation of climate adaptation strategies is to find a measure of the timeframe over which the climatic stressor can be expected to put substantial pressure on ecosystems. We additionally suggest that reference to recent conditions is likely to be of greater relevance for policy issues than reference to the natural variability of the system prior to any anthropogenic influence (i.e. to the pre‐industrial period). Because of rapid socio‐economic development in recent decades, current social and business structures are aligned with contemporary variability. Furthermore, many areas of the world's oceans are already experiencing climate change with quantifiable impacts on marine resources (i.e. Frusher *et al*., 2014) and have consequently begun adapting to this change. Consequently, a period characterized by variability unperturbed by anthropogenic forces is already in the distant past for many areas and not at all reflective of the conditions and resources that present‐day societies are reliant upon.With these considerations in mind, we select the period 1990–2009 as a baseline and define the range of ‘baseline variability’ as an averaged value over this period plus or minus two standard deviations from the mean (for a normal distribution, this encompasses 95% of the variation). We further assume that a climate stressor would be more likely to apply pressure to an ecosystem if it regularly falls outside of this range of variability during the 21st century. As a measure of such behaviour, we use the number of years in decades 2010–2019 and 2020–2029 when the climate stressor is outside of the range of its baseline variability (1990–2009). We categorize deviation from baseline variability as ‘substantial’ if, in any given decade, 5 of 10 years are outside of this range.

A suggested concept of the baseline variability vs. trend originates from the studies Henson *et al*. (2010) and Beaulieu *et al*. (2013) of the satellite‐derived and modelled values of chl‐a. These studies suggested that the magnitude of natural variability in primary production and chlorophyll is larger than, or similar to, the climate change signal in contemporary, short (10 years) satellite record (Henson *et al*., 2010). Similarly, Boyd *et al*. (2008) suggested that change in the Southern Ocean primary production could not be separated from its high natural variability until approximately 2040.

## Results

### SST linear trends in CMIP5 and NEMO models

**Figure 1:**
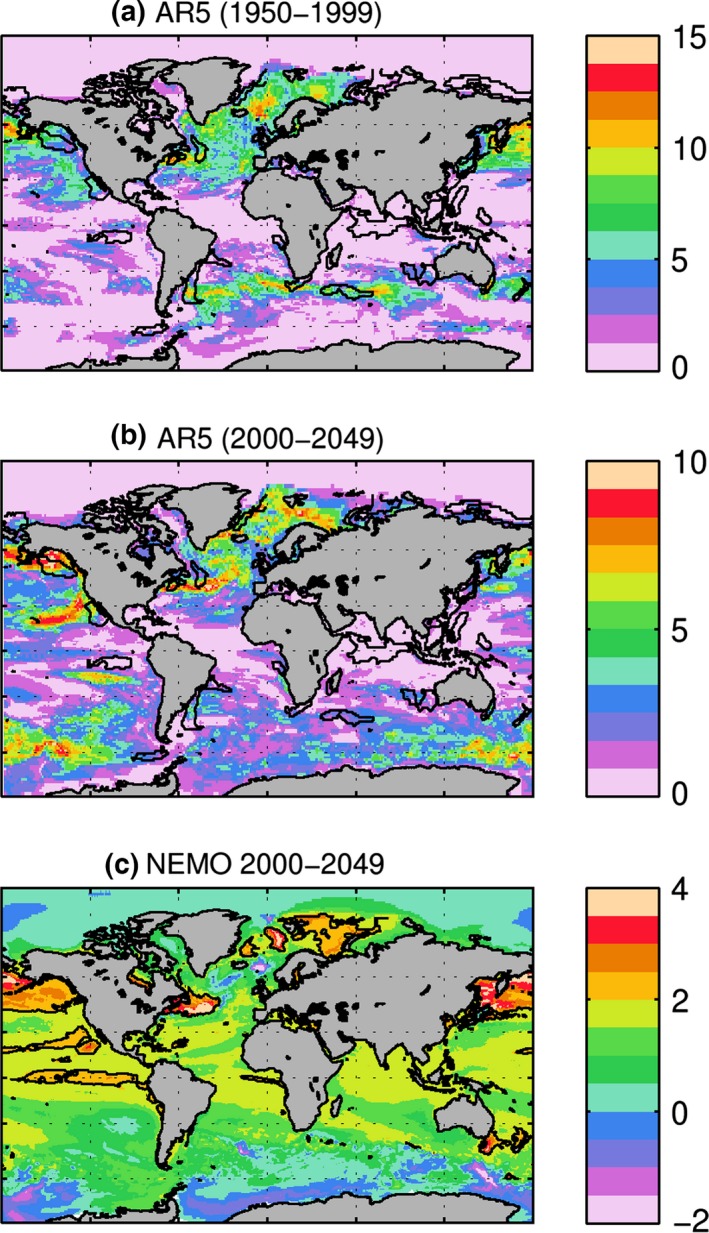
(a) Overlap in occurrence of hotspots based on the historical (1950–1999) linear SST trend from 23 models used in CMIP5. The colour bar represents the number of models with a hotspot at the pixel location. Hotspots are identified as 10% of the fastest warming areas. (b) the same as (a) but for 2000–2049 period under RCP8.5 scenario; (c) SST linear trend in NEMO for 2000–2049 (°C per 50 yr). Black contours on subplots a, b show hotspots identified in the same way by HP14 on the basis of historical observations. Black contours on subplot c show hotspots identified in the same way by HP14 on the basis of NEMO linear trend for 2000–2049.

On the basis of historical sea surface temperature (SST) trends for the period 1950–1999, HP14 identified 24 marine hotspots representing the upper 10% of areas affected by ocean warming. Using the same approach, they showed that 19 of the 24 historical hotspots were identified in at least one of the six climate models from the CMIP3 archive (IPCC TAR). Following HP14, we repeated the analysis for the 23 models from the CMIP5 archive (Table 1). With the increase in horizontal model resolution from 2 to 3° in CMIP3 to ~1° in the majority of the CMIP5 models (Taylor *et al*., 2012), it is natural to expect improvements in the models’ ability to reproduce spatial patterns of the fastest historical warming. The frequency of hotspot occurrence in the CMIP5 models (number of models showing existence of a hotspot in a grid point) is shown in Fig. 1a. In agreement with Wu *et al*. (2012), we note a higher consistency for models to reproduce hotspots in the areas of the western boundary current extensions (Brazil, Kuroshio, Gulf Stream, Agulhas and East Australian currents). Models are also consistent in reproducing enhanced warming in the area of the Californian upwelling, and the subarctic Pacific and East Greenland currents. However, hotspots in tropical and equatorial areas (including the Mozambique Channel and Indian hotpots analysed in this study), as well as those in the high Arctic, are not generally reproduced by the models. These are the areas where higher resolution is particularly important to reproduce the key features of ocean dynamics. Thus, we find that CMIP5 models reproduce the locations of warming hotspots more consistently than those from CMIP3, and it is to be expected that increases in resolution in CMIP6 models will with further improve hotspot representation in models.

**Table 1:** Models from the CMIP5 archive http://cmip-pcmdi.llnl.gov/cmip5/data_portal.html used in calculation of the marine hotspots

Modelling Centre (or Group)	Institute ID	Model name	Number of ensemble runs used	References
Community Earth System Model Contributors	NSF‐DOE‐NCAR	CESM1(BGC)	1	Moore *et al*. (2004)
Centre National de Recherches Météorologiques/Centre Européen de Recherche et Formation Avancée en Calcul Scientifique	CNRM‐CERFACS	CNRM‐CM5	1	Voldoire *et al*. (2013)
NOAA Geophysical Fluid Dynamics Laboratory	NOAA GFDL	GFDL‐ESM2G GFDL‐ESM2M	1 1	Dunne *et al*. (2012, 2013)
Met Office Hadley Centre (additional HadGEM2‐ES realizations contributed by Instituto Nacional de Pesquisas Espaciais)	MOHC (additional realizations by INPE)	HadGEM2‐CC HadGEM2‐ES	3 4	Collins *et al*. (2011)
Institut Pierre‐Simon Laplace	IPSL	IPSL‐CM5A‐LR IPSL‐CM5A‐MR IPSL‐CM5B‐LR	4 1 1	Seferian *et al*. (2013)
Max‐Planck‐Institut für Meteorologie (Max Planck Institute for Meteorology)	MPI‐M	MPI‐ESM‐MR MPI‐ESM‐LR	1 3	Ilyina *et al*. (2013)
Norwegian Climate Centre	NCC	NorESM1‐ME	1	Tjiputra *et al*. (2013)

Will these hotspot areas persist into the future? The frequency of hotspot occurrence in CMIP5 models for the period 2001–2050 (RCP8.5 scenario) is shown in Fig. 1b. The models are consistent in projecting the Gulf Stream and Kuroshio extensions and the subarctic Pacific as remaining as fast‐warming areas, and they are joined by the prominent additions of the Barents Sea and the California current. At least one‐third of the models show the Brazil and East Australian currents as future hotspots, while the Indian, Mozambique Channel and South African hotspots persist into the future in only 1–2 models. Similar trends were noted by HP14 in the lower resolution CMIP3 models.

The future linear SST trends found in NEMO are in general agreement with those from the CMIP5 runs (Fig. 1c). Of the five focal hotspots, East Australia shows the fastest warming trend of up to 8 °C for the 2001–2050 time period examined, followed by the South African hotspot (in particular the area of Agulhas retroflection) with warming of up to 4 °C across the 50‐yr period. The warming trends across the rest of the hotspots are approximately 3 °C over the 50‐yr period (cf. 1.3 °C across the 50 years period global mean).

### Ocean circulation: model validation and future projections

As suggested by Wu *et al*. (2012), some of the fastest warming observed is associated with the shift of and/or intensification of boundary currents. The key importance of circulation for potential changes in ecosystem functioning across various regions of the ocean has been highlighted by several authors, for instance Sorte (2013), Roughan *et al*. (2011), Buchanan *et al*. (2014) and Wassmann *et al*. (2015). Particularly, important factors are the pivotal role of circulation in setting species range limits, and in limiting or facilitating species redistribution in a changing climate. Consequently, changes to ocean circulation are one of the key climate change‐induced stressors of ocean ecosystems, and it is therefore critical to assess how well models can reproduce key circulation features if their projected changes are to be judged reliable.

Decadal‐averaged (2000–2009) modelled surface ocean circulation and satellite‐derived velocities are shown in Fig. S1. Decadal‐averaged values have been chosen to focus on persistent circulation features that are not obscured by short‐term variability such as mesoscale eddies. The comparison shows good agreement between observed and modelled surface circulation features, with a correlation coefficient (*r*) of 0.69 for the global domain. For each of the hotspots, a range of patterns are observed as described below.

The main circulation feature of the Brazilian hotspot is the Brazil current, a western boundary current that flows southward as part of the subtropical gyre of the South Atlantic Ocean. The model reproduces this current well (Fig. S1c, d), with a r of 0.66.

The Agulhas current, one of the strongest currents in the world oceans, is the main feature of the South African hotspot. This is a western boundary current of the southern Indian Ocean subtropical gyre that flows south‐west along the east coast of Africa. Towards the southern tip of Africa, it separates from the coast, looping anticlockwise as the Agulhas retroflection and feeding back into the Indian Ocean (e.g. Beal *et al*., 2011). The model reproduces the location and strength of the Agulhas retroflection well (with a r of 0.68), although it is narrower in the model with underestimated variability at its periphery due to model resolution not being fully eddy‐resolving (see Discussion). On the western coast of Africa, the model clearly shows the Benguela Current, a northward flowing ocean current that forms the eastern portion of the South Atlantic Ocean gyre (Fig. S1e, f), although this current is probably too narrow to be clearly defined in the AVISO data product.

In the Mozambique Channel hotspot (Fig. S1g, h), surface circulation is dominated by the South equatorial current that feeds the East Madagascar current and Mozambique current (Tomczak & Godfrey, 2003). Here, agreement between modelled and observed currents is weaker than in the previous hotspots, with a *r* of 0.52. Although the model reproduces the strength and location of the currents, they are also narrower than observed, again due to an insufficient transfer of horizontal energy by mesoscale eddies. This is especially pronounced in the Mozambique Channel which is characterized by frequent occurrence of anticyclonic eddies along the western flank of the channel (e.g. Quartly & Srokosz, 2004) which the model under‐represents.

Circulation of the northern Indian Ocean is characterized by the seasonally varying surface currents driven by the Indian monsoon. In particular, in winter, the Bay of Bengal is characterized by strong anticyclonic circulation turning into a weak cyclonic one in summer (Potemra *et al*., 1991). In such a seasonally varying case, comparison of the annual mean circulation is less informative, and in Fig. S1i, j for illustration, we present December‐averaged surface circulation instead. This clearly shows the western boundary current along the eastern coast of India both in the model and in AVISO (*r* for this month is 0.62).

The East Australian hotspot (Fig. S1k, l) is dominated by the East Australia current, another western boundary current. The path of this current from Australia to New Zealand is known as the Tasman Front and is characterized by strong meanders and eddies. The model reproduces the location of the boundary current (*r* 0.69); however, it underestimates the strength of the mesoscale variability.

**Figure 2:**
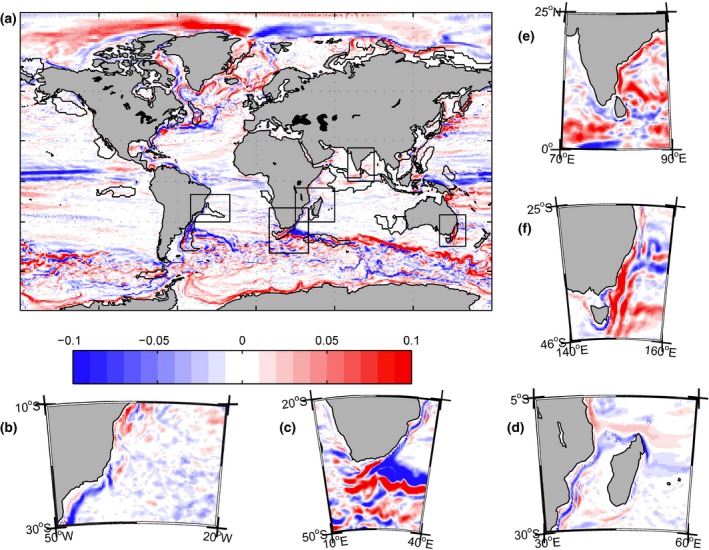
Relative deviation of the decadal‐averaged surface current speed of 2000–2009 from 2050 to 2060. (a) global distribution; magnified view for five regional hotspots: Brazilian (b) South African (c), Mozambique Channel (d), Indian (e) and East Australian (f).

Figure 2 shows the relative deviation of the decadal‐average current speed of 2050–2059 (‘2050s’) from that of the period 2000–2010 (‘2000s’). The results show the following general features: a weakening of the Brazil current and its shift eastward in the northern part of the area and westward in the southern part of the area (Fig. 2b); a south‐east shift and intensification of the Agulhas retroflection (Fig. 2c); a weakening of the Mozambique current (Fig. 2d); intensification of the circulation in the Bay of Bengal (Fig. 2e); and a southward shift and intensification of the East Australia Current. In the context of global changes in circulation (Fig. 2a), the South African and Australian hotspots are amongst the areas experiencing the strongest shift and/or intensification of the dominant surface currents globally, similar to the Gulf Stream, Kuroshio and south most part of the Brazil current/Subtropical Convergence (outside the Brazil hotspot region considered here). Although our results point towards future changes in circulation, further in‐depth Lagrangian study is required to estimate the impact such changes may impose on ocean ecosystems in terms of changes in the nutrient pathways, connectivity and migration/extinction of species with planktonic phases.

### Upper mixed layer depth, dissolved inorganic nitrogen and primary production

#### Model validation

We chose the maximum annual depth of the upper mixed layer (UML; based on monthly mean values) as a convenient measure of the state of water column stratification and exchange with deeper layers (e.g. Popova *et al*., 2006). The modelled global distribution and regional hotspot patterns of maximum UML depth, as well as its climatology from the World Ocean Atlas (WOA, Locarnini *et al*., 2006; Antonov *et al*., 2006), are shown in Fig. S2. The five hotspots of focus here span areas from the equatorial (e.g. Indian hotspot), with typically low‐annual variability and maximum UML <50 m, to high latitudes (e.g. the southern part of the east Australian hotspot), where winter mixing is substantial and penetrates below 300 m. The model broadly reproduces these contrasting regimes. The largest discrepancies between the model and the observed WOA climatology occur at the east Australian hotspot where the model underestimates the depth of winter mixing at its southern edge (Fig. S2k, l). Similarly, the model underestimates winter mixing in the southern part of the South African hotspot (Fig. S2e, f). In spite of the model capturing general patterns of UML variability, some regional discrepancies remain, and their attribution to a particular feature (or features) of the model is not straightforward. Nonetheless, UML dynamics are critical for those of the modelled ecosystem, and their representation should thus remain a focus for improvement as climate models develop.

Modelled and climatological annual mean concentrations of dissolved inorganic nitrogen (DIN), averaged over the top 100 m of the water column, are shown in Fig. S3. The model has a tendency to underestimate DIN concentration in oligotrophic areas, and in particular for the Brazilian, Indian and Mozambique Channel hotspots. These three hotspots are more oligotrophic in the model than observed climatology suggests, with modelled annual mean values underestimated by a factor of two. In the cases of the Brazilian and Mozambique Channel hotspots, this underestimation can be attributed, at least partially, to the underestimated depth of winter mixing (cf. Fig. S2). Underestimated nutrient concentration in the subtropical gyres is a well‐known problem of global and basin‐scale models (e.g. Levy *et al*., 2001). It typically results from both physical issues such as insufficient resolution (e.g. Popova *et al*., 2006) and omitted biogeochemical factors such as nitrogen‐fixing phytoplankton and the lower carbon to nitrogen ratios that occur in these oligotrophic regions (e.g. DeVries & Deutsch, 2014; Teng *et al*., 2014). Although the model utilized in this study is substantially improved in resolution relative to the CMIP5 models, this resolution is still inadequate to fully describe mesoscale and submesoscale processes that act as components of vertical nutrient supply (e.g. Levy *et al*., 2012). By contrast, the South Africa and east Australian hotspots are located outside of oligotrophic areas and DIN concentrations are reproduced well compared to the climatology (Fig. S3).

Model and satellite‐derived (see Yool *et al*., 2013a; for methodology) water column‐integrated primary production are shown in Fig. S4. In this case, the satellite‐derived values should be taken as guidance only as these are estimates derived from the simple average of three algorithms developed to relate ocean colour to phytoplankton productivity and the uncertainty of these estimates is substantial especially in the shelf regions (as per Yool *et al*., 2013a). The five focus hotspots span a wide range of productivity regimes from highly oligotrophic (<<0.5 g C m^−2^ day^−1^; substantial areas of the Brazilian and Mozambique Channel hotspots) through moderately productive (0.5–0.8 g C m^−2^ day^−1^; the Indian and east Australia hotspots) to some of the most productive ecosystems of the world (>0.8 g C m^−2^ day^−1^; part of the South Africa hotspot). In general, the model reproduces this range well, although as a result of underestimated DIN concentrations in the subtropical gyres, primary production there is also underestimated. This underestimation most clearly manifests itself in the Brazilian and Mozambique Channel hotspots (Fig. S3c, d, g, h) where primary production is about half that of the satellite‐derived estimates.

Again, due to limited resolution and missing shelf processes, the model has a tendency to underestimate productivity in shelf regions. The Indian hotspot, with its shelf‐enhanced primary production, presents an example of this. In contrast, the Brazilian hotspot presents an interesting example where the model does reproduce shelf‐enhanced productivity possibly because of the large‐scale upwelling associated with the Brazil Current (e.g. Campos *et al*., 2000). The model shows higher than observed primary production in the most southerly areas of the east Australian and South African hotspots as it tends to locate a transition zone to the low productive Southern Ocean further south than is indicated by the observations. Overestimated primary production is driven by the higher than observed DIN in these hotspots, which in turn is probably a result of overestimated vertical diffusivities in the Southern Ocean (Yool *et al*., 2013a,b).

#### Future projections

**Figure 3:**
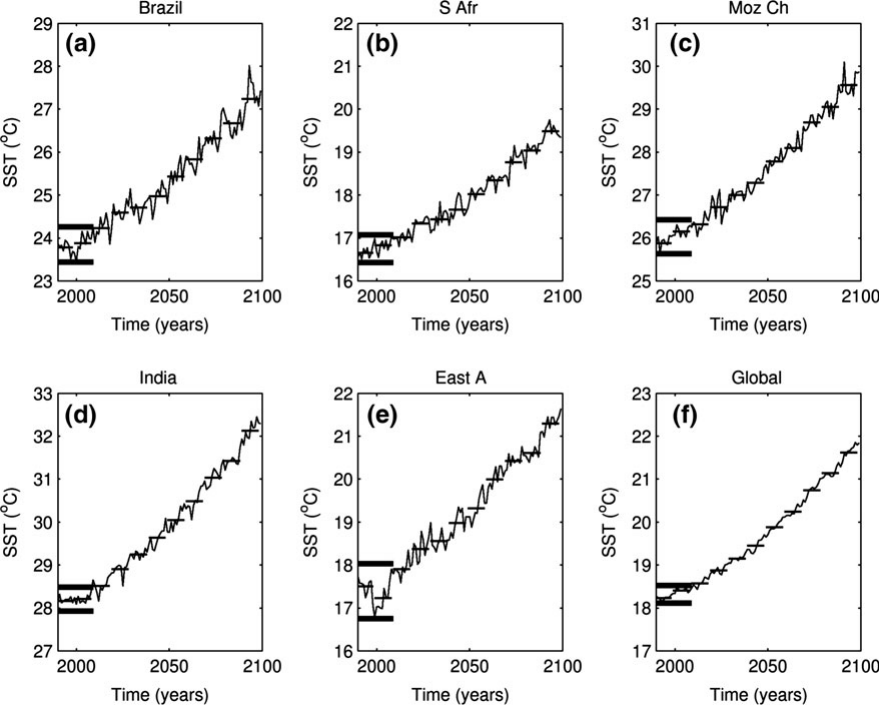
Annual mean SST (°C) for the period 1990–2099 averaged over the hotspot areas shown as black rectangles on panels a and b of Figs S2–S4. Decadal‐averaged values shown as thick horizontal lines. Range of recent variability (1990–2010, see text) shown as thin horizontal lines.

**Figure 4:**
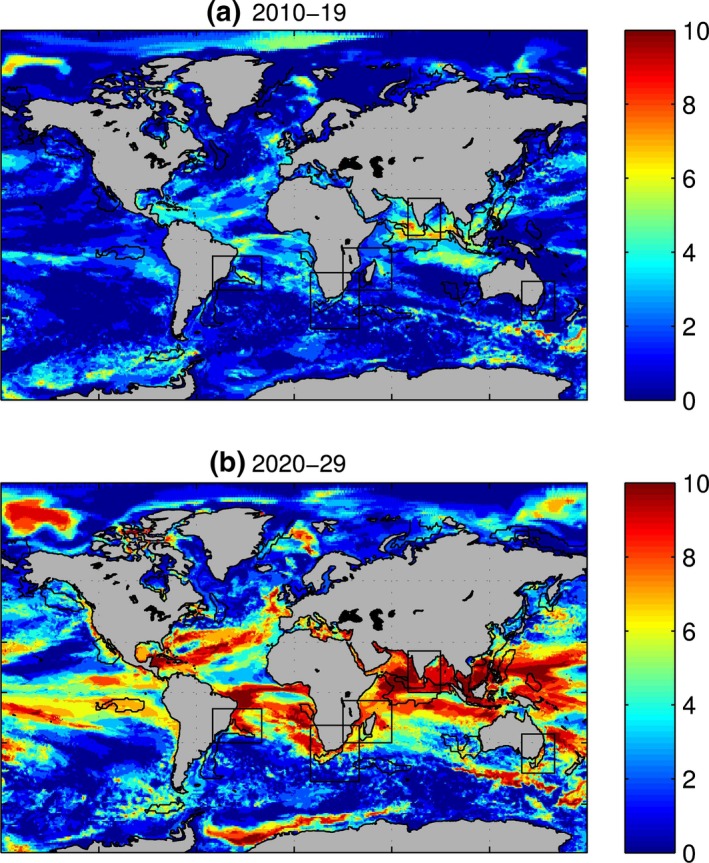
Number of years in a decade 2010–2019 (a) and 2020–2029 (b) when annual SST falls outside of the range of its recent variability.

Next, we analyse projected future changes of these model characteristics averaged over hotspot areas. Figure 3 shows model annual and decadal mean SST for the period 1990–2099, averaged for the hotspot areas. Strong increases in SST are evident for all areas even against a background of interannual variability. From 2000 to 2099, SSTs typically change by around 3–4 °C across all of the hotspots examined, with the South African hotspot showing the smallest increase of around 3 °C (Fig. 3b) and the east Australian and Indian hotspots showing the greatest increases, in excess of 4 °C. The hotspot of eastern Australia has the strongest baseline variability (17.4 ± 0.6 °C, Fig. 3a), while the Indian Hotspot (Figs 3d) is characterized by the lowest baseline variability (28.3 ± 0.3 °C). Consequently, in the current decade (2010–2019, Fig. 4a), eastern Australia is experiencing a rate of change similar to its baseline variability, with SST showing values outside of this range in the decade 2020–2029, while the Indian hotspot already manifests SST values outside of the range of natural variability in the current decade. Over decade 2020–2029 (Fig. 4b), all five hotspots experience a substantial change in SST over at least part of their area.

**Figure 5:**
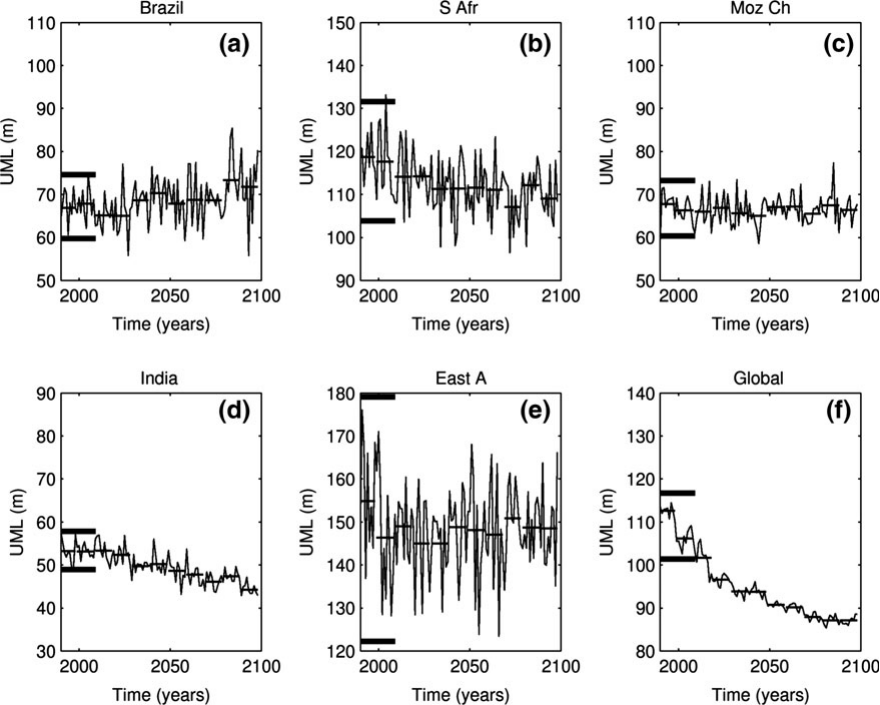
Same as Fig. 3 for maximum UML depth (m).

**Figure 6:**
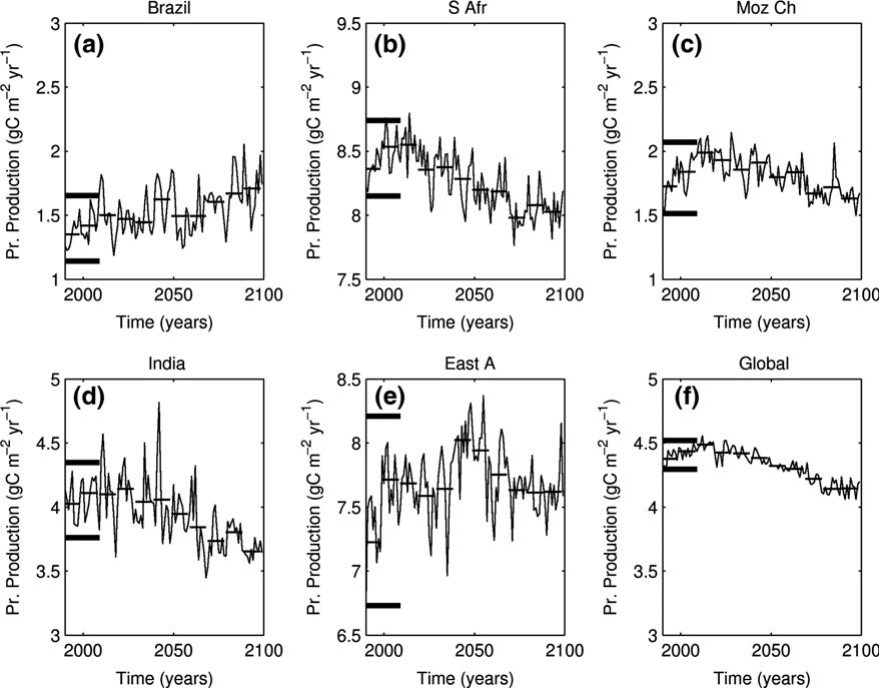
Same as Fig. 3 for annual primary production (g C m^−2^ yr^−1^).

We analyse the other three indicators (stratification, nitrate and primary production) in a similar manner with the time evolution for 1990–2099 presented in Figs 5 and 6, Fig. S5. Unlike SST, these stressors manifest strong interannual variability but do not show substantial deviation from their baseline variability at any time over the century. The only hotspot where stratification (maximum UML depth) shows substantial shallowing is the Indian hotspot (Fig. 5d), where shallow values consistently occur after 2050. Interestingly, and in contrast with other regions, stabilization of stratification in this area is accompanied by an increase in nitrate. This probably points towards changes in horizontal advection, rather than stratification, as being the main driver of nutrient content in this area.

We examine the number of years in decades 2010–2019 and 2020–2029 when primary production is outside of the range of its baseline variability (Fig. S6). Unlike SST (cf. Fig. 4 and Fig. S6), primary production does not a show substantial deviation from its baseline variability in any of our five focus hotspots nor, in fact, in almost any of the other hotspots reported in HP14, with the exception of the Indo‐Chinese hotspot. Over the global ocean, areas with the strongest signal in primary production are situated in the Gulf Stream, the western equatorial Pacific, the equator‐ward flanks of the South Pacific subtropical gyre and the marginal ice zones of the Southern Ocean. Over the global scale, decoupling of the fastest warming areas from the areas of strongest changes in primary production is not surprising. While increases in SST generally work towards stabilization of stratification, and thus a reduction in nutrient supply, it will only have a pronounced effect on primary production in the areas that are already limited by nutrients and where concentrations are dominated by vertical supply mechanisms. Similarly, the details of carbonate chemistry and the geographical pattern of oceanic CO_2_ uptake mean that ocean acidification and SST impacts are not coincident in space.

### Future changes in ocean acidification

Ocean acidification – the consequence of oceanic uptake of anthropogenic carbon dioxide from the atmosphere – is now widely recognized as a major stressor on ocean ecosystems (e.g. Gruber *et al*., 2012; Bopp *et al*., 2013; Cyronak *et al*., 2014). Ocean acidification is expected to impact key physiological and ecological processes of organisms, with consequences for ecosystems in which they occur and benefits obtained from them by society (Gattuso *et al*., 2014). Environmental conditions in which aragonite (the more soluble of two biogenic forms of calcium carbonate) becomes undersaturated are projected to first occur in polar regions (e.g. Popova *et al*., 2014), and even under the RCP8.5 scenario are unlikely to become a threat in the focus areas in the current century (Yool *et al*., 2013b). To illustrate this, the first occurrence of monthly mean undersaturated surface waters in respect to aragonite is shown in Fig. S7, and this is shown both in terms of years of the 21st century and in values of atmospheric CO_2_. For ocean acidification to impact ecosystems in our focus hotspots, atmospheric pCO_2_ should substantially exceed 1000 ppm (Popova *et al*., 2014).

Although Fig. S7 is focused on surface conditions, undersaturation on the seabed of shelf waters is also important, as many important and vulnerable species live in benthic habitats. The first occurrence of undersaturation in the shelf bottom waters generally follows the same large‐scale patterns of temporal progression as on the ocean surface. However, it may occur earlier in the areas affected by upwelling (e.g. Gruber *et al*., 2012). In our focus hotspots, the model shows onset of shelf bottom water undersaturation towards the end of the century in the Indian hotspot (northern part of the Bay of Bengal), the east Australian hotspot (Bass Strait) and the South African hotspot (western coast of Africa).

### Future changes in oxygen minimum zones (OMZ)

Reduced concentrations of dissolved oxygen (O_2_) occur for a number of reasons. In coastal zones, they may result from eutrophication, which causes increased surface productivity and then increased oxygen consumption in interior waters (e.g. Gilbert *et al*., 2010). Another cause is ocean warming, which decreases the solubility of oxygen in surface waters and effectively decreases the ocean inventory of dissolved O_2_. Coupled to this is reduced interior ventilation because of circulation or stratification changes, which tend to decrease supply of dissolved O_2_ to the ocean's interior. Warmer surface temperatures also tend to increase remineralization rates of sinking organic material, with the result that the majority of oxygen consumption can be focused into a narrower, shallower band. Below a certain hypoxic threshold (50–80 mmol m^−3^; Bopp *et al*., 2013) serious damage to ecosystems can be expected. Modelled vertical extent of oxygen minimum zones (OMZs) for decade 2000–2009 and its future changes by decade 2050–2060 are shown in Fig. S8. Figure S8b shows how changes to the OMZ manifest as characteristic ‘stripes’ of alternating expansion and contraction. These stripes are a result of shifts in the positions of the main currents that define the boundaries of the OMZs (Brandt *et al*., 2015). As can also be seen in Fig. S8, OMZs are most pronounced in eastern boundary upwelling areas, systems that none of our focus hotspots belong to. The only focus area with an OMZ is the Indian hotspot where the modelled region of hypoxia expands into the Bay of Bengal. By the decade 2050–2060, the OMZ in this region expands into the Arabian Sea, but at the same time, it weakens in the northern part of the Bay of Bengal.

## Discussion

### Main climatic stressors in the marine warming hotspots

The concept of marine warming hotspots suggested by HP14 on the basis of historical SST data builds on a considerable literature defining hotspots for biodiversity (e.g. Myers, 2003). Analysing climate change impacts in such hotspots – regions that are experiencing high rates of change in this dominant climatic driver – may be useful in science–policy partnerships to facilitate an increase in the capacity of local communities to adapt to climate related change (e.g. Frusher *et al*., 2014; de Sherbinin, 2014; HP14; Pecl *et al*., 2014). In this study, we have analysed key climate change‐driven ecosystem stressors in five temperature‐defined marine hotspots in the Southern Hemisphere. Our aim has been to translate the richly detailed output of a climate model into a form which can readily be used to help guide research directions and decision‐making processes in the relevant regions.

In our assessment of climatic stressors on ocean ecosystems, we suggest that the main question that should be asked is when a stressor begins to fall outside of either the range of natural (before the anthropogenic influence; Landres *et al*., 1999) or a baseline (a given fixed period) variability, and if such an occurrence is part of a consistent trend. To quantify such conditions, here we use a criterion of two standard deviations from the mean for the period 1990–2010. If a stressor consistently falls outside this range, this criterion strongly suggests that the system is undergoing significant change relative to the baseline. The analysis of SST, stratification (expressed as maximum depth of winter mixing), nitrate concentration within top 100 m and water column primary production show that the rise of SST is unequivocal in all hotspots areas (Fig. 3), although its impact on ecosystems might manifest itself differently (depending upon factors such as local habitats and biota). The fastest rise of SST amongst the focal hotspots is in the eastern Australia hotspot (Fig. 1c); however, this area is also characterized by strong interannual variability (Fig. 3a). Will the historical hotspots identified in HP14 remain the fastest warming areas in the future? CMIP5 model SST trends to 2050 generally agree that the fastest warming areas will carry on being associated with the western boundary currents. Further, because of polar amplification, the Northern Hemisphere will continue to have both a greater number and more intense hotspots than the Southern Hemisphere (e.g. Pithan & Mauritsen, 2014). However, east Australia remains a hotspot across the majority of the models, and there are indications that the Brazilian hotspot will also persist into the future. Nevertheless, all five focus hotspots remain the fastest warming areas of the Southern Hemisphere.

Contrary to the unequivocal trend in SST, the responses to climate change of the three other main drivers (stratification, DIN and primary production) are weak due to their strong natural variability.

Ocean deoxygenation is widely considered as one of the major human‐induced stressors of ocean ecosystems (e.g. Bopp *et al*., 2013). It is anticipated to accelerate in the next century as a result of the reduced solubility of oxygen (a temperature effect), reduced ocean ventilation (a stratification effect), increasingly shallow remineralization of sinking organic material (a temperature effect) and an increase in productivity in some areas. That said, dynamic effects can counterbalance these factors and may even lead to contractions of OMZs (e.g. Deutsch *et al*., 2014). The major large‐scale OMZs are associated with the eastern boundaries of the tropical Pacific, Atlantic and northern Indian oceans, and their recent expansion and intensification has been detected in observations (Stramma *et al*., 2008). As the distribution of OMZs is set by a balance between the vertical profile of organic material remineralization, ocean ventilation and circulation, reproducing OMZ distribution and variability in numerical models is challenging (cf. Stramma *et al*., 2012). Nevertheless, our model reproduces the general geographical distribution of the OMZs and generally forecasts their expansion into the future (Yool *et al*., 2013b). However, the reader should note that our analysis of OMZs is limited as MEDUSA does not include either denitrification or nitrogen fixation, both of which would affect local DIN concentrations. Only one of our focus hotspots, the Indian hotspot (Fig. S8), is located within a large‐scale OMZ, in the Northern Indian Ocean (Gilly *et al*., 2013). By decade 2050–2060, the OMZ in this region is forecast to expand into the Arabian Sea, but at the same time, it weakens in the northern part of the Bay of Bengal. This illustrates that, although climate change is global, regional impacts need not track average change (e.g. Cocco *et al*., 2013).

Using the saturation state of the biomineral aragonite (Ω), we showed that ocean acidification driven by the ocean uptake of anthropogenic CO_2_ is unlikely to become a major threat in the five focus hotspot areas in this century (assuming RCP8.5 scenario; Fig. S7). Largely due to naturally low Ω, the Arctic Ocean is the basin first projected to manifest surface undersaturation in respect to aragonite, and this has already occurred in some areas (e.g. Bates *et al*., 2011). The onset of undersaturation in the Arctic is followed by that in the Southern Ocean, with the widespread surface undersaturation in the most southerly areas propagating northward by around 2050, and by areas of the eastern boundary upwelling systems which typically manifest widespread surface undersaturation towards the end of the century (e.g. McNeil & Matear, 2008; Yool *et al*., 2013b).

### Ocean circulation as a stressor of marine ecosystems

Ocean warming, acidification, deoxygenation and reduced productivity (resulting from increased stratification) are widely considered to be the major stressors to ocean ecosystems induced by emissions of CO_2_ (e.g. Bopp *et al*., 2013). However, a stressor overlooked in this list is the change in ocean circulation in response to climate change. Strong changes in the intensity and position of the western boundary currents are already observed (Wu *et al*., 2012), and the consequences of such changes for ecosystems are beginning to emerge (e.g. Matear *et al*., 2013). In this respect, the east Australian hotspot is probably the most pronounced example of the impact that circulation changes can have on marine ecosystems. Various lines of evidence point towards the intensification of the East Australian Current (e.g. Hill *et al*., 2008; Johnson *et al*., 2011; Buchanan *et al*., 2014) and link major changes in ecosystem dynamics of the region to the direct impact of advection (e.g. Ling *et al*., 2009; Thompson *et al*., 2009; Johnson *et al*., 2011; Suthers *et al*., 2011; Robinson *et al*., 2015). These impacts are above and beyond the indirect impacts driven by circulation change accelerating SST rise.

The example of the east Australian hotspot also illustrates that, while SST remains one of the most straightforward and easy‐to‐observe indicators of climate change, it may be providing us with a limited picture. It draws attention to the fact that some regions with naturally high SST variability – and, thus, less clear SST trends – are also experiencing well‐documented changes in ecosystem dynamics associated with ocean circulation change. An example of a similar region outside of the scope of this study, but also characterized by a strong natural variability of oceanographic cycles, is the Galapagos archipelago, situated at the confluence of five ocean currents. This area has already experienced major biodiversity losses as a result of the synergistic impacts of changes in circulation and major oceanographic characteristics likely altered by climate change, as well as overfishing (Edgar *et al*., 2009).

In this paper, we have presented projected changes of the main surface currents affecting five focus hotspots. In particular, we noted a weakening and shift of the Brazil current; a south‐east shift and intensification of the Agulhas retroflection; a weakening of the Mozambique current; intensification of the seasonally reversible circulation in the Bay of Bengal; and a southward shift and intensification of the East Australia Current. In the context of global changes of the circulation (Fig. 2a), South African and Australian hotspots are amongst the areas experiencing the strongest shift/intensification of the dominant surface currents, similar to the Gulfstream, Kuroshio and south most part of the Brazil Current (outside of the Brazil hotspot considered here). The indirect impact of advection was accounted for in this study through projected changes in the main stressors, such as SST or productivity. However, estimating the direct impact of changing circulation is a much more difficult task that that can best be studied using Lagrangian approaches (e.g. Popova *et al*., 2013; Kendall *et al*., 2016). We diagnosed potential changes in the strength and location of the main currents. However, this work is only a first step in understanding projected changes in ocean circulation, and future work that examines the roles of changing transport and connectivity in‐depth will be necessary to properly characterize the significance of this driver for change in ocean ecosystems.

### Role of model resolution in future climate stressor projections

Earth system models include atmosphere, ocean, cryosphere, and terrestrial and marine biota components and have been developed to investigate whole‐Earth impacts of future climate change. Against a backdrop of constantly increasing computing power, these models have correspondingly increased in the spatial detail that they can resolve. The last two decades have seen progress from typically 2 to 3° horizontal resolution in IPCC AR4 (Hasumi, 2014) to around 1° in AR5 (Hasumi, 2014), and it is anticipated that 1/4° models currently under development will be used in the expected AR6. That said, it is important at the outset to acknowledge the extreme computational cost of such models, and a number of modelling strategies have been developed to manage or constrain these costs. One such approach is to run ocean‐only models under atmospheric forcing derived from extant climate change runs. This allows simulations of the ocean to achieve high spatial resolution at lower cost, albeit at a price of omitting feedbacks with the rest of the Earth system.

In this study, we employ such an approach and drive our ocean model at a resolution of 1/4° with forcing derived from an existing IPCC RCP8.5 simulation (Yool *et al*., 2013a,b). We show that this resolution allows us to achieve a certain regional realism at a spatial scale appropriate to marine hotspots, mostly because of a better representation of ocean circulation and, specifically, ocean western boundary currents. Such increased realism of ocean circulation is paramount for hotspot analysis, as the root of fast warming in these marine areas is change in the variability or location of local western boundary currents (Wu *et al*., 2012). Equally important is the improved representation of upwelling areas, although the resolution of the overlying atmosphere is of high relevance in the case of coastal upwelling (Hasumi, 2014). Upwelling zones are also crucial in the analysis of marine hotspots as they are often areas of decreased oxygenation and increased ocean acidification, two important stressors of marine ecosystems (Bopp *et al*., 2013), although not ones that particularly impact our five focus hotspots except for the Indian Ocean.

Figure S9 illustrates the change in modelled representation of the Agulhas current and its retroflection as model resolution increases from 1° to 1/4° (employed in this study) to 1/12°, as compared to satellite‐derived AVISO data. Although the largest step‐change in improved realism of boundary current strength and location is undoubtedly made when the resolution changes from 1° to 1/4°, circulation variability on its periphery and, more generally, within the gyres only becomes realistic as the resolution increases to 1/12°, at which point eddies are not just permitted, but are resolved. Thus, although an era of CMIP6 models of 1/4° resolution promises to approach regional realism at sub‐basin scale, there remains a strong motivation for the more computationally efficient forced ocean models to be employed for analyses of climate change impacts on ecosystems. Undoubtedly, regional downscaling can achieve much greater resolution and, consequently, representation of regional details (e.g. Matear *et al*., 2013) including the dynamics of inner shelf and coastal zone features which are important when large‐scale climatic drivers are being translated into the local impacts (Holt *et al*., 2015). However, as Fig. S9 illustrates, downscaling can only be as successful as the lower resolution, basin‐ or global‐scale model from which it is downscaled. Additionally, for regional cross‐comparison studies such as this one, the use of a high‐resolution, global‐scale model remains a compellingly self‐consistent way forward, even if its resolution does not permit all mesoscale or submesoscale features. Substantial improvements in the representation of ocean circulation translate into better performance of marine biogeochemistry. Figure S10 shows annual mean primary production (year 2000) for 1° and ¼° models alongside a satellite‐derived estimate. The following main features are noteworthy: an increase in productivity at the centres of oligotrophic gyres by a factor of 2‐3; improvement in the spatial distribution of primary production in the Bay of Bengal and in the vicinity of Agulhas Retroflection; more pronounced local productivity maxima associated with the Brazil current. However, at the same time, we note a worsening of the agreement in the Mozambique Channel, where underestimated primary production at 1° resolution is even lower in the ¼° resolution version of the model. A detailed discussion of performance issues related to increased resolution is beyond the scope of this manuscript; however, they are discussed in part elsewhere (Yool *et al*., 2015).

In part because of high computing cost, future projections of the climatic stressors presented here are made on the basis of a single run of a single model, and thus, estimates of uncertainty are not yet possible. Although ensembles of Earth System models are available in the CMIP5 archive, their resolution is insufficient to address mechanisms behind the hotspot drivers associated with the ocean circulation and in the majority of cases with the western boundary currents. It is anticipated that CMIP6 will see increases in model resolution – reaching 1/4° in some models – and this will allow uncertainty estimates to be made. The alternative approach of estimating uncertainty on the basis of multiple runs of our model is limited because of the high cost of global runs at this resolution.

### Relating climate change models to the needs of adaptation policy

The likely impacts of climate change on fisheries and fishing communities are being given increasing attention (e.g. Allison *et al*., 2009; Cochrane *et al*., 2009; Gasalla & Diegues, 2011), but there is still only limited practical experience in adaptation to climate change in coastal communities (e.g. van Putten *et al*., 2013; Shelton, 2014; Shyam *et al*., 2014), as well as an urgent need to improve and test the theories and practices that underpin existing efforts (Pecl *et al*., 2014). To develop such theories and design practical solutions, a clear picture of how climate change will alter multiple environmental properties in the ocean is needed. In particular, what will such changes mean for those coastal communities that are highly dependent on marine resources?

One of the main obstacles to accommodating climate change into future management strategies is the complexity of information provided by models and the difficulty in relating large‐scale climate trends to local impacts. There are no simple generic approaches to adaptation in fisheries and fishing communities: each case needs to be assessed through integrated planning to achieve clearly defined adaptation objectives (Porter *et al*., 2014), and research is required for understanding and predicting species‐specific responses to climate change in order to predict future stock responses (Pecl *et al*., 2014). Evaluating the vulnerability of societies to climate change impacts requires both (a) knowledge of the natural and climate‐induced variability of relevant environmental factors and (b) the likely consequences of such changes to local communities. As such, simplified relevant information from climate models is needed to facilitate links between both climate and socio‐economic research, and the science that informs resource management and strategy (Boyd *et al*., 2011; Hobday *et al*., 2013, 2016).

One possible approach for summarizing the main results of this study is illustrated in the Appendix S1 and Fig. S11. While such a summary is inevitably rudimentary, it presents the impacts of the stressors on marine ecosystems in a format that aims to facilitate the necessary socio‐economic analysis (Hobday *et al*., 2015). Our analyses indicate that adaptation to climate change impacts on coastal fisheries and fishing communities will be required in all five hotspots. Simplified information from climate models, as presented here, will assist in both climate and socio‐economic research and facilitate the integrated approaches that are required to build resilience in the most vulnerable social–ecological systems.

## Supporting information


**Fig. S1.** Decadal averaged (2000–2009) surface current speed (m s^−1^) from AVISO (a) and NEMO model (b). Subplots c‐l show magnified view of panel a (left column) and panel b (right column) for five regional hotspots considered in this paper and shown as black rectangles on panels (a) and (b).

**Fig. S2.** Annual maximum UML depth based on the monthly mean values (m). Global model results for the decade 2000–2009 (a), climatology (b); Subplots c‐l show magnified view of panel a (left column) and panel b (right column) for five regional hotspots considered in this paper and shown as black rectangles on panels a and b: Brazilian (c, d) South African (e, f), Mozmbique Channel (g, h), Indian (i, j) and East Australian (k, l).

**Fig. S3.** Same as Fig. S2 for the annual mean dissolved Inorganic Nitrogen averaged over top 100 m (DIN, mmol N m^−3^).

**Fig. S4.** Same as Fig. S2 for the annual mean water column primary production (g C m^−2^ day^−1^).

**Fig. S5.** Same as Fig. 3 for annual DIN averaged over top 100 m (mmol N m^−3^).

**Fig. S6.** Number of years in a decade 2010–2019 (a) and 2020–2029 (b) when annual mean primary production falls outside of the range of its recent variability.

**Fig. S7.** The first occurrence of a monthly mean undersaturated surface waters in respect aragonite in years (a) and level of atmospheric pCO_2_ (b).

**Fig. S8.** Vertical extent of oxygen minimum (O_2_ < 50 mmol O_2_ m^−3^) zones (m) for the decade 2000–2009 (a) and changes in vertical extent between decade 2050–2059 and 2000–2009 (m).

**Fig. S9.** Decadal averaged (2000–2009) surface current speed (m s^−1^) from NEMO model at resolution 1 (a) 0.25° (b), 1/12° (c) and from AVISO (d) for the South African hotspot.

**Fig. S10.** Annual averaged primary production (g C m^−2^ yr^−1^) from model at resolution 1 (a) and 0.25 (b), satellite‐derived estimates (c).

**Fig. S11.** A simplified diagram presenting the main climatic‐driven risk factors on marine ecosystems for each of the hotspots for decades 2020–2029 and 2080–2089 in a format that aims to facilitate the necessary socio‐economic analysis.

**Appendix S1** Climate risk factors for marine ecosystems.
