# Supplementary figures and images for: From global to regional and back again: common climate stressors of marine ecosystems relevant for adaptation across five ocean warming hotspots
Source: Glob Chang Biol. 2016 Mar 21;22(6):2038–53. doi: 10.1111/gcb.13247 (PMC4999053; doi:10.1111/gcb.13247)

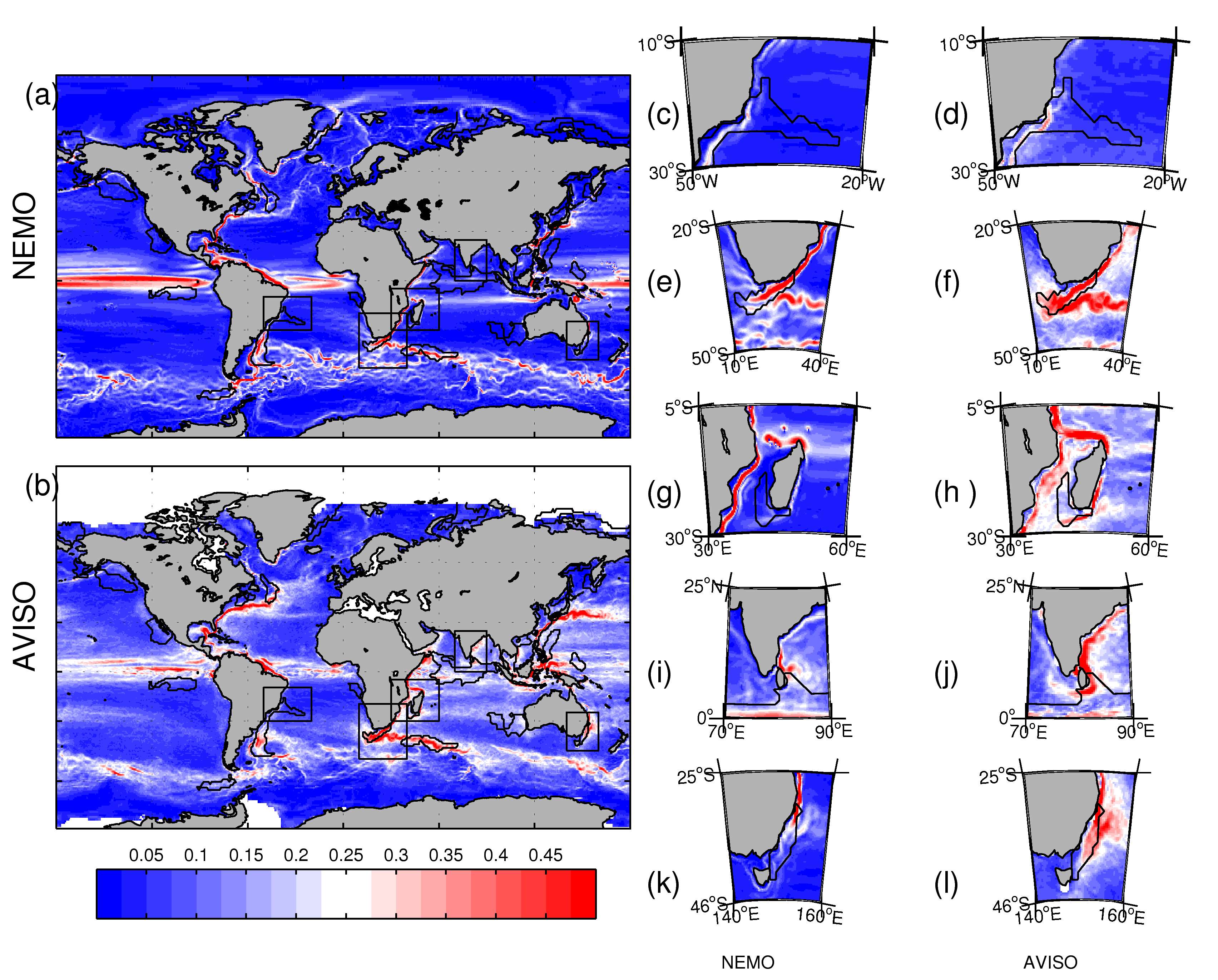

Supplement: Supplementary file 1 — Fig. S1. Decadal averaged (2000–2009) surface current speed (m s−1) from AVISO (a) and NEMO model (b). Subplots c‐l show magnified view of panel a (left column) and panel b (right column) for five regional hotspots considered in this paper and shown as black rectangles on panels (a) and (b). [file GCB-22-2038-s001.jpg]

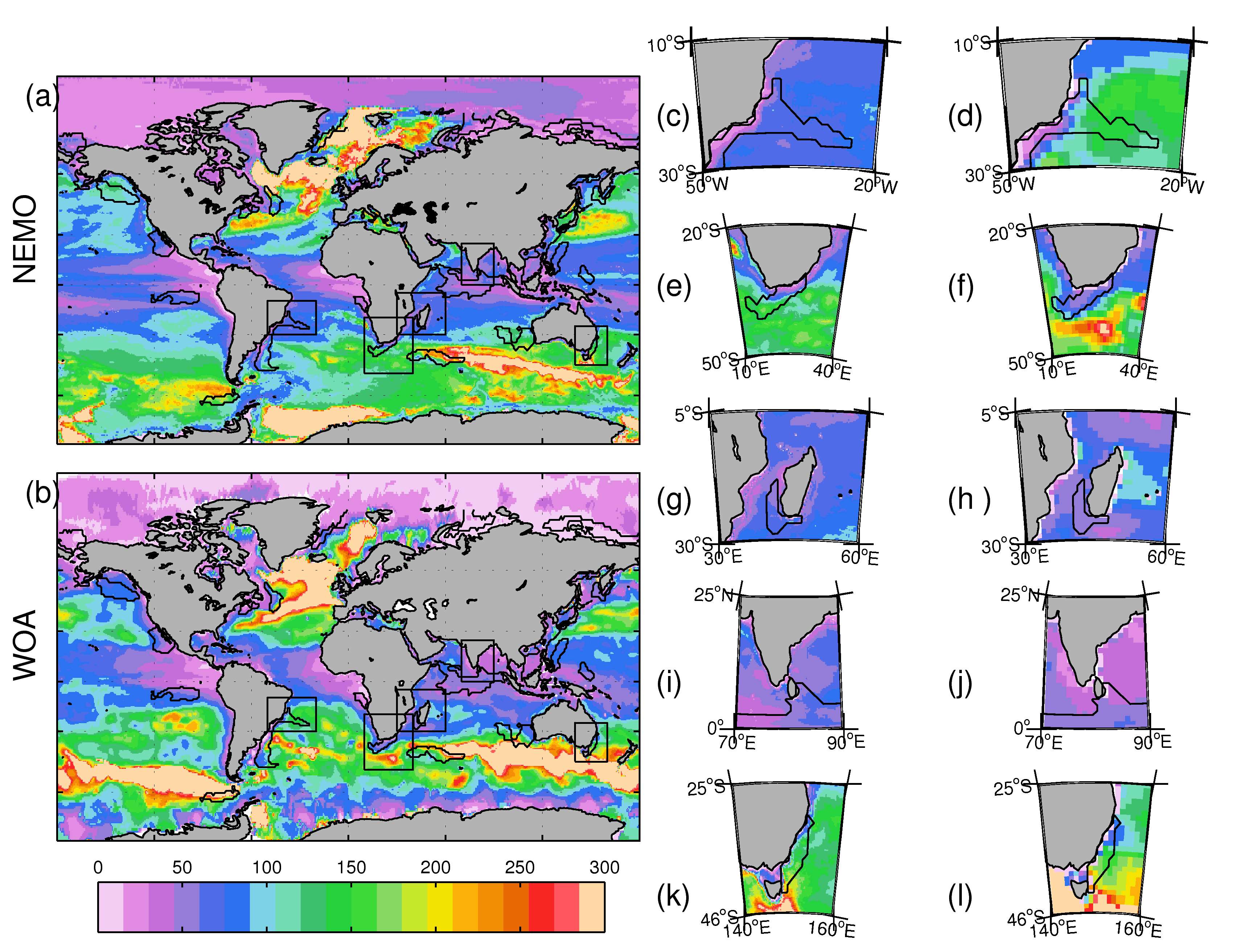

Supplement: Supplementary file 2 — Fig. S2. Annual maximum UML depth based on the monthly mean values (m). Global model results for the decade 2000–2009 (a), climatology (b); Subplots c‐l show magnified view of panel a (left column) and panel b (right column) for five regional hotspots considered in this paper and shown as black rectangles on panels a and b: Brazilian (c, d) South African (e, f), Mozmbique Channel (g, h), Indian (i, j) and East Australian (k, l). [file GCB-22-2038-s002.jpg]

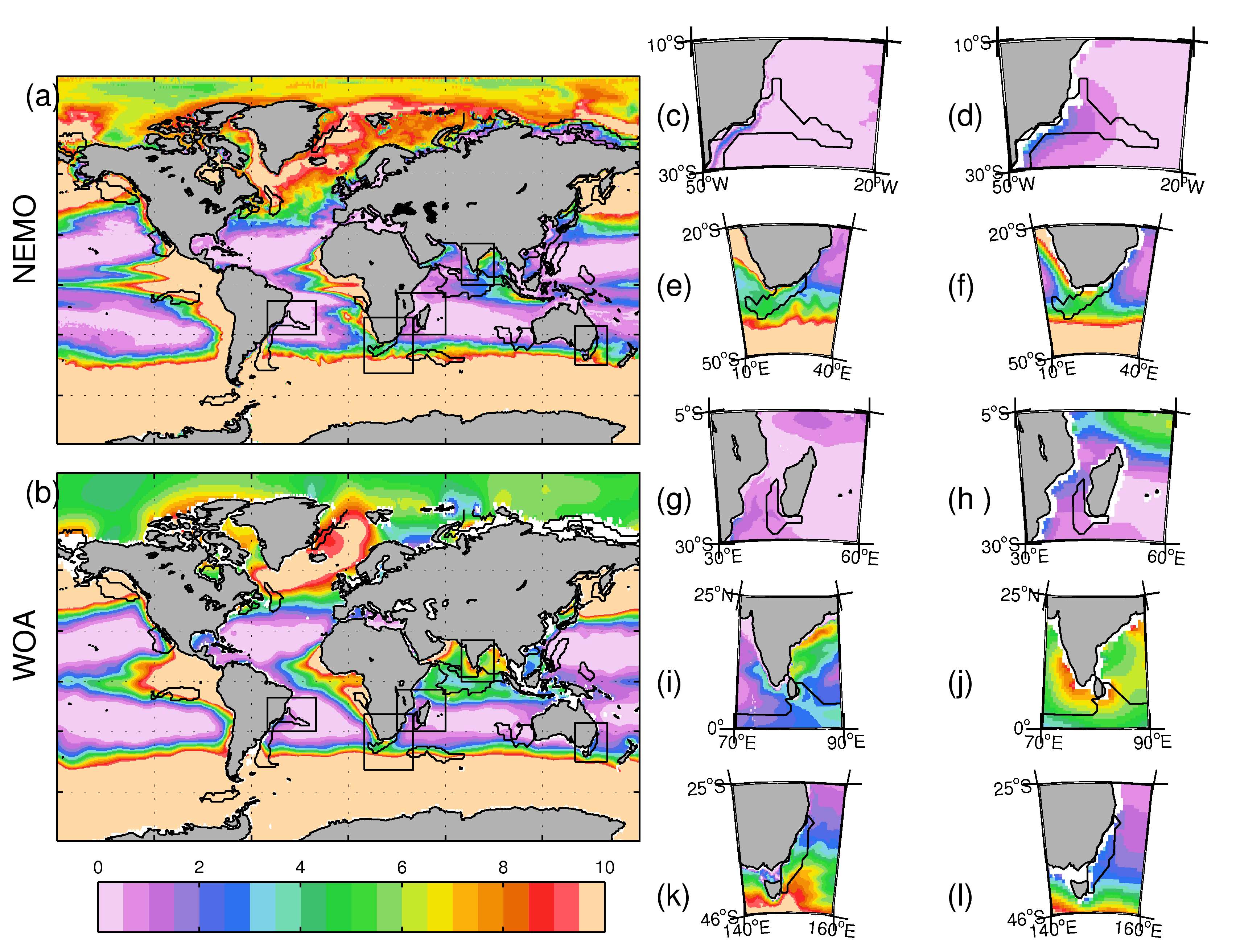

Supplement: Supplementary file 3 — Fig. S3. Same as Fig. S2 for the annual mean dissolved Inorganic Nitrogen averaged over top 100 m (DIN, mmol N m−3). [file GCB-22-2038-s003.jpg]

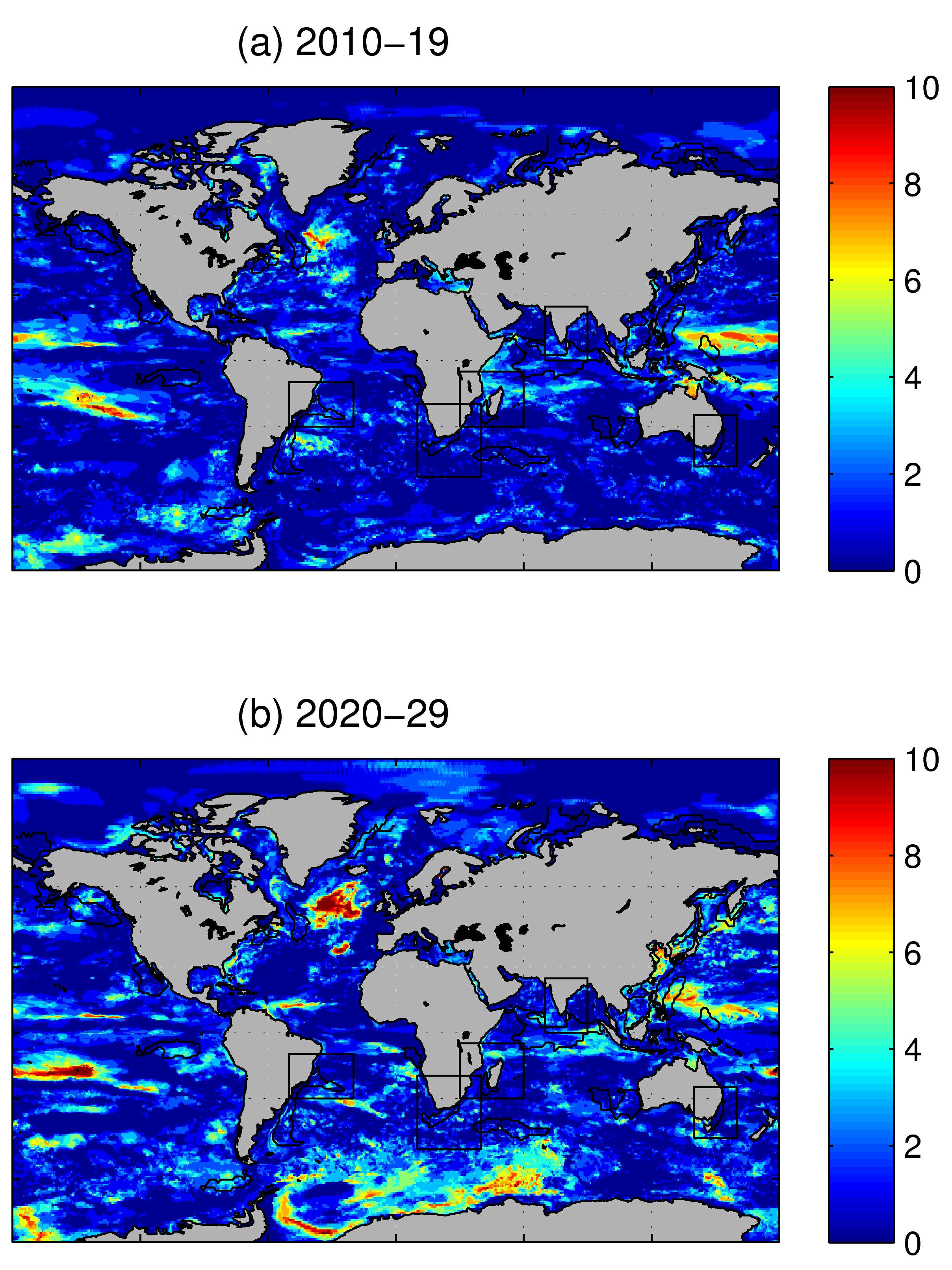

Supplement: Supplementary file 6 — Fig. S6. Number of years in a decade 2010–2019 (a) and 2020–2029 (b) when annual mean primary production falls outside of the range of its recent variability. [file GCB-22-2038-s006.jpg]

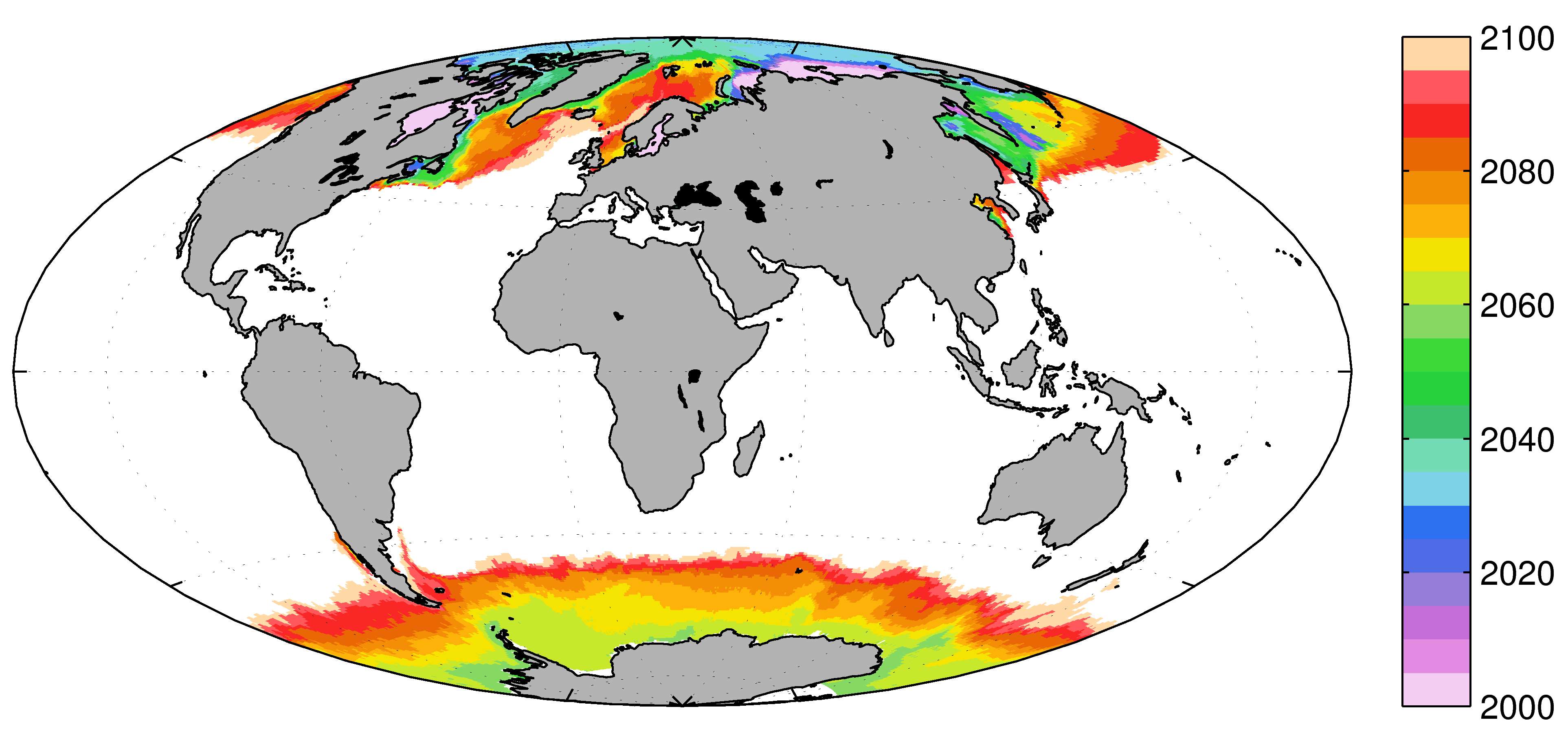

Supplement: Supplementary file 7 — Fig. S7. The first occurrence of a monthly mean undersaturated surface waters in respect aragonite in years (a) and level of atmospheric pCO2 (b). [file GCB-22-2038-s007.jpg]

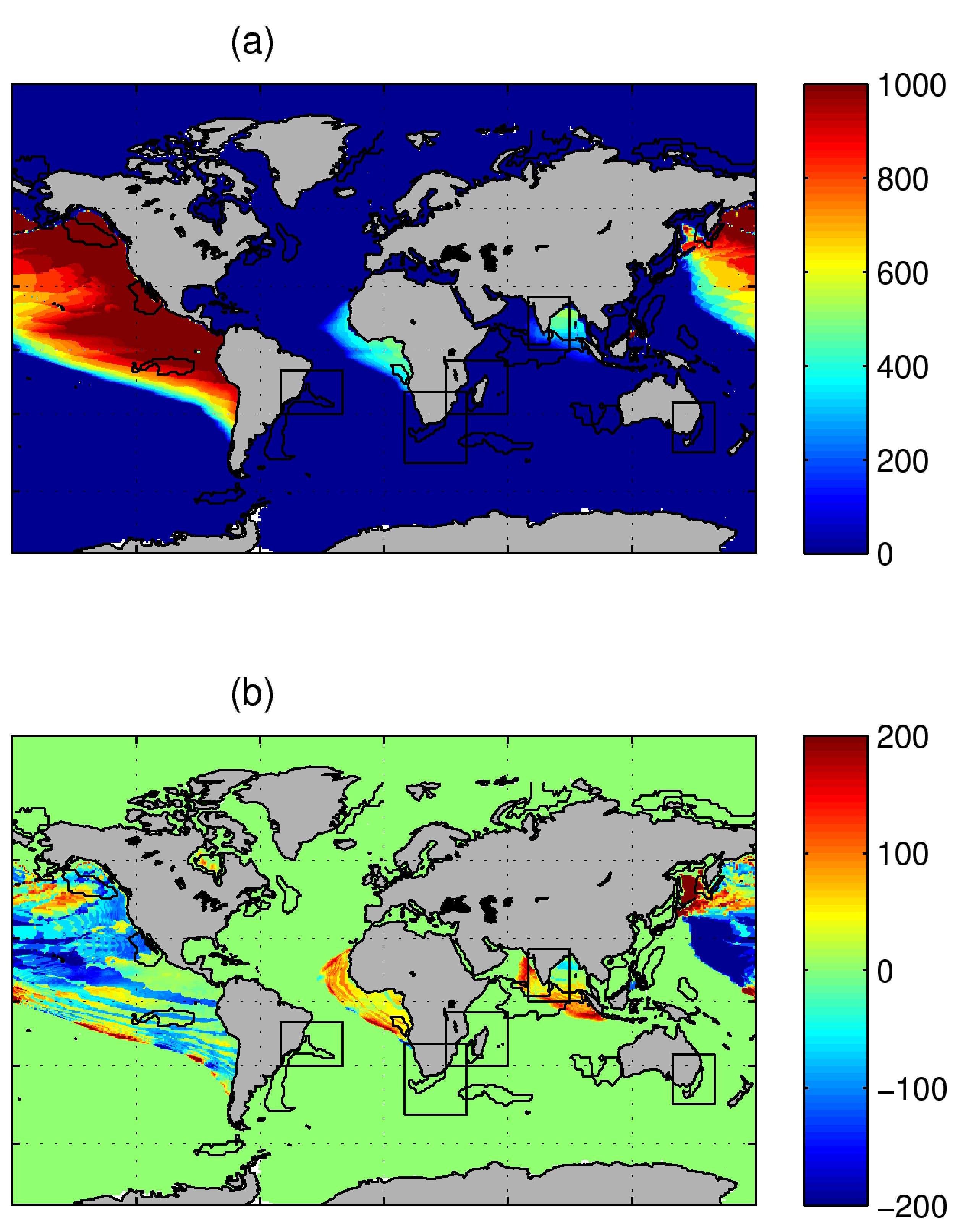

Supplement: Supplementary file 8 — Fig. S8. Vertical extent of oxygen minimum (O2 < 50 mmol O2 m−3) zones (m) for the decade 2000–2009 (a) and changes in vertical extent between decade 2050–2059 and 2000–2009 (m). [file GCB-22-2038-s008.jpg]

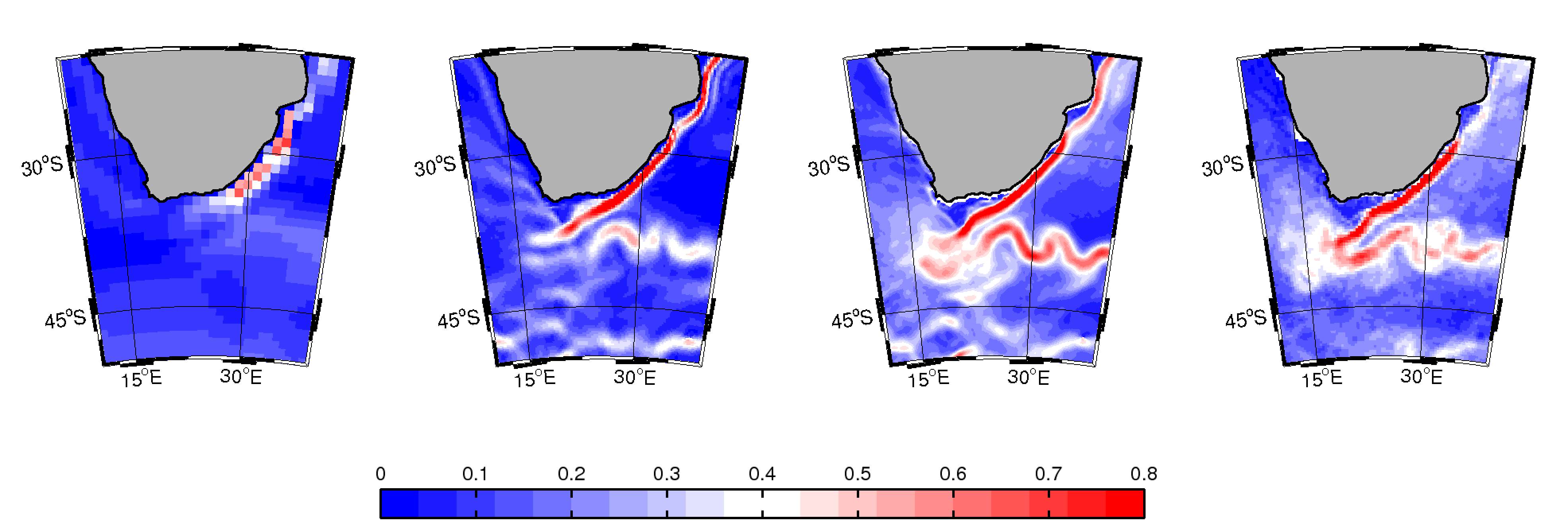

Supplement: Supplementary file 9 — Fig. S9. Decadal averaged (2000–2009) surface current speed (m s−1) from NEMO model at resolution 1 (a) 0.25° (b), 1/12° (c) and from AVISO (d) for the South African hotspot. [file GCB-22-2038-s009.jpg]

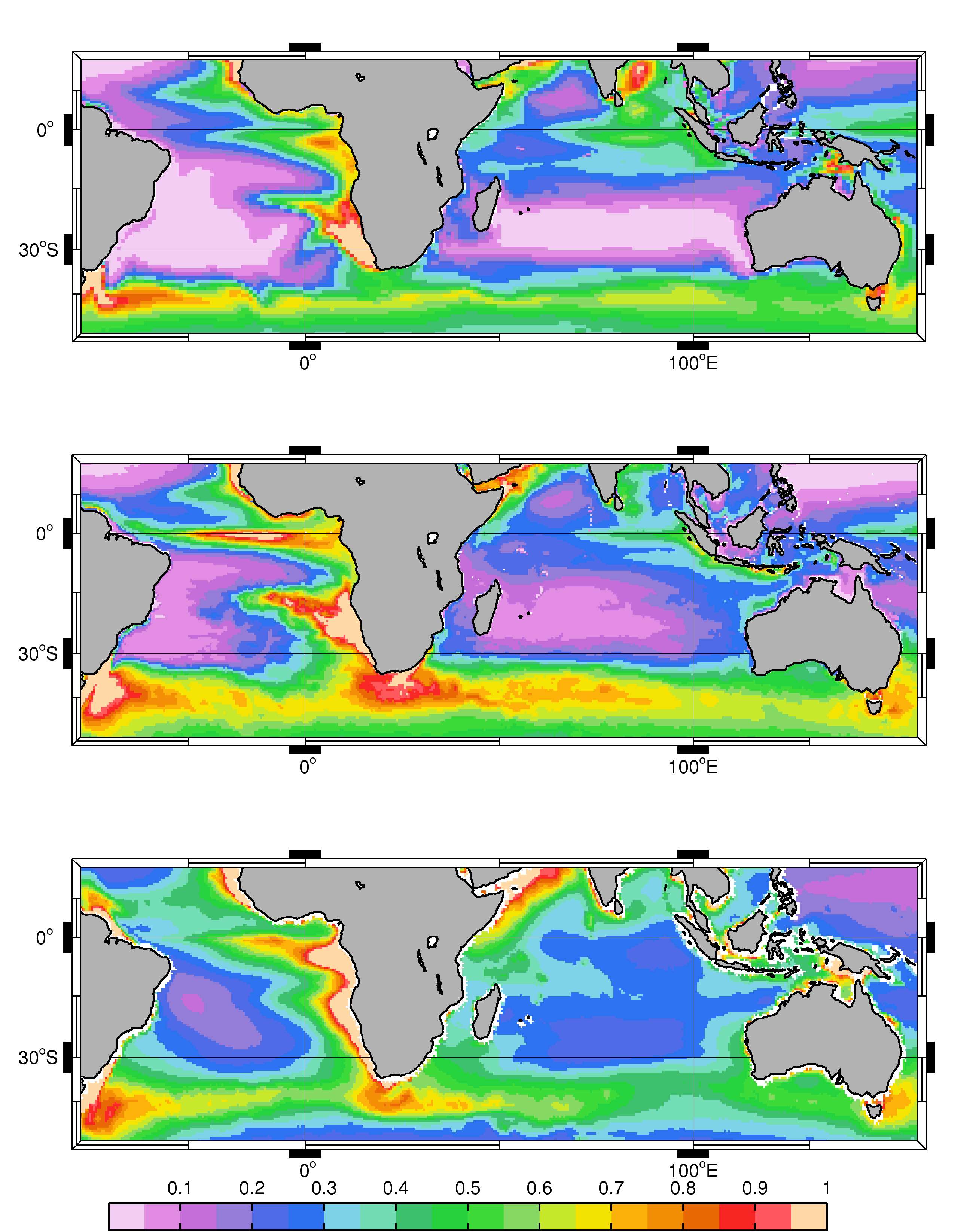

Supplement: Supplementary file 10 — Fig. S10. Annual averaged primary production (g C m−2 yr−1) from model at resolution 1 (a) and 0.25 (b), satellite‐derived estimates (c). [file GCB-22-2038-s010.jpg]

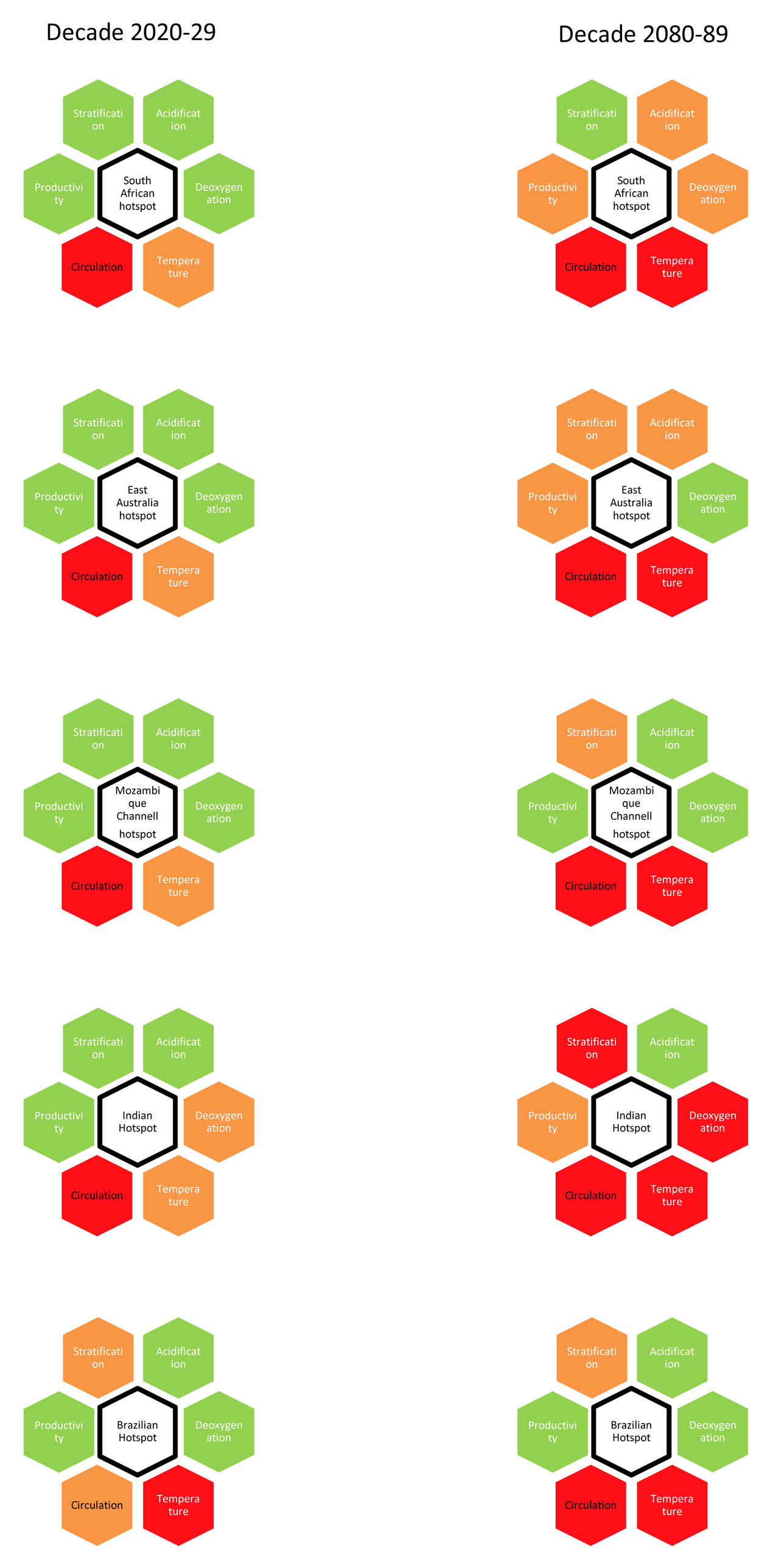

Supplement: Supplementary file 11 — Fig. S11. A simplified diagram presenting the main climatic‐driven risk factors on marine ecosystems for each of the hotspots for decades 2020–2029 and 2080–2089 in a format that aims to facilitate the necessary socio‐economic analysis. [file GCB-22-2038-s011.jpg]
