# Supplementary material for: From global to regional and back again: common climate stressors of marine ecosystems relevant for adaptation across five ocean warming hotspots
Source: Glob Chang Biol. 2016 Mar 21;22(6):2038–53. doi: 10.1111/gcb.13247 (PMC4999053; doi:10.1111/gcb.13247)
Supplement: Supplementary file 12 — Appendix S1 Climate risk factors for marine ecosystems. [file GCB-22-2038-s012.docx]

**Supporting information**

S1. Climate risk factors for marine ecosystems

In order to facilitate dialog between climate scientists and policy makers we have adopted the following approach to summarise our study results into an easy-to-assimilate form.

We identify the following six climatic risk factors of marine ecosystems: i) warming of the ocean; ii) decline of marine primary production; iii) change in ocean stratification; iv) deoxygenation; v) ocean acidification; vi) change in ocean circulation. The impacts of ocean stratification and primary production are not decoupled since water column stratification regulates the nutrient supply to the euphotic zone and, thus, ocean productivity. However, our consideration of the ocean stratification as a separate stressor in addition to primary production reflects its additional potential impact on planktonic community composition (e.g. Margalef, 1978; Bopp et al., 2005).

In our assessment of climatic stressors on ocean ecosystems, we would argue that the main questions which should be asked are: i) when does a stressor begin to fall outside of the range of baseline variability, and ii) is such an occurrence part of a consistent trend. Since the stressors we have identified affect ecosystems in different ways, here we evaluate these questions using a series of quantitative criteria.

In the cases of temperature, primary production and stratification, we use a criterion of two standard deviations from the mean for the period 1990-2010. Specifically, for each decade (2010-2019, 2020-2029, …, 2080-2099) we count the number of years when annual mean SST, annual mean primary production and annual maximum upper mixed layer depth falls outside of this two standard deviations window. If this number is five years or above, we assume that this climate stressor constitutes a risk factor and is equal 1, alternatively it is 0.

For ocean acidification, we determine the decade in which either surface or near bottom shelf waters (restricted to the ocean depth less than 220m) become undersaturated in respect to aragonite (a more soluble form of calcium carbonate) for at least one month per year (cf. Popova et al., 2014). Starting from this decade, the acidification risk factor becomes 1 since atmospheric CO2 increase is both the dominant factor affecting omega, and constantly increases under RCP 8.5 – effectively, once a hotspot is classified as experiencing acidification stress, it remains this way.

For oxygen minimum zones, the risk factor is set to 1 should decadal averaged oxygen minimum zones either expand their thickness in a given grid point (relative to the 1990-2010 averaged values) or appear in places where they were previously absent.

Finally, for ocean circulation changes, the risk factor is set to 1 if a change in the decadal averaged surface velocity relative to the 1990-2000 period exceeds 5 cm s^-1^. Note that this high threshold value is chosen in order to reflect changes only in the main ocean currents such as western boundary and equatorial currents, and Antarctic Circumpolar Current.

The risk level associated with each of the stressors is assigned as follows. Low: climate change stressor is 0 over the hotspot area; Medium: the stressor is 1 in some grid points and can be considered as a warning signal; High: the stressor is 1 over a substantial (>10%) part of the area.

Summary of the main climatic-driven risk factors for marine ecosystems for each of the hotspots for decades 2020-29 and 2080-89 is shown on the Figure S11. This figure will assist resource managers in each region in gaining an understanding of the expected changes in the main environmental drivers of marine resources. Our current work provides information on the magnitude and rate of change in these key environmental parameters, guiding resource managers and researchers in investigating those resources that can be expected to be significantly affected (Pecl et al., 2014). Advance warning of impending changes to the distribution, timing or abundance of marine resources offers the opportunity for adaptation in the human system to such change.
